# Kyste hydatique pulmonaire bilateral

**DOI:** 10.11604/pamj.2016.24.280.7700

**Published:** 2016-07-28

**Authors:** Meriem Lahroussi, Wiam El Khattabi, Nihal Souki, Hasna Jabri, Hicham Afif

**Affiliations:** 1 Service des Maladies Respiratoires, Hôpital 20 Août, Casablanca, Maroc

**Keywords:** Kyste hydatique pulmonaire, hydatidose, cestodose, Hydatid cyst, lung, bilateral forms, surgery

## Abstract

L'hydatidose est la cestodose humaine la plus fréquente. La localisation pulmonaire vient au 2^ème^ rang des localisations viscérales après la localisation hépatique. L'imagerie joue un rôle important dans le diagnostic et le bilan d'extension. Elle renseigne sur le nombre, le siège, l'aspect et la taille des kystes hydatiques pulmonaires. Le but de notre observation consiste à soulever une stratégie diagnostique hiérarchisée devant une présentation radiologique particulière d'un kyste hydatique pulmonaire bilatérale.

## Abstract (en)

Hydatid disease is the most common human cestodosis. Pulmonary localization is the 2^nd^ largest visceral locations after liver localization. The imaging plays an important role in the diagnosis and staging. It provides information on the number, the seat, the appearance and size of pulmonary hydatid cysts. The aim of our observation is to raise a particular pulmonary hydatid cyst bilateral radiological presentation.

## Introduction

La maladie hydatique est une infestation parasitaire endémique. Elle est encore un vrai problème de santé publique dans le monde. La fréquence de l'hydatidose pulmonaire bilatérale peut varier de 4 à 26%. Le diagnostic de la polykystose pulmonaire repose sur des critères cliniques, des tests sérologiques et des techniques d'imagerie. Le traitement curatif ne peut être que chirurgical.

## Patient et observation

**Figure 1:**
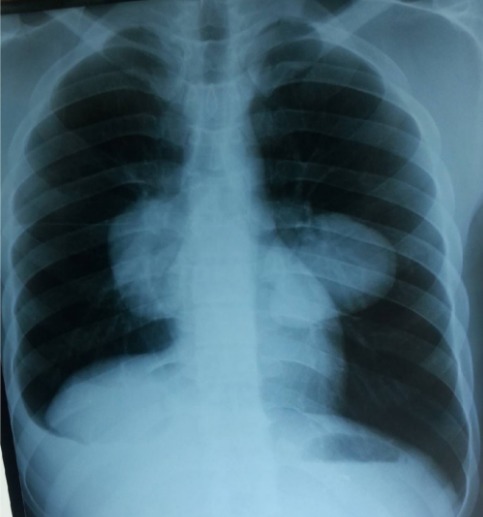
La radiographie thoracique de face en inspiration montre deux opacités arrondies à projection hilaires avec un aspect surélévation de la coupole diaphragmatique droite et un emoussement du cul de sac costodiaphragmatique droit

Un patient âgé de 23 ans, plombier de profession depuis 6 ans, tabagique chronique à 13 paquets années et sans antécédent pathologique particulier. La symptomatologie clinique remontait à un mois avec l'installation progressive d'une douleur thoracique bilatérale en point de côté non irradiante et une hémoptysie de faible abondance sans autres signes respiratoires. À l'admission, l'examen clinique a trouvé un patient en bon état général, apyrétique, normotendu et eupneique. L'examen pleuro-pulmonaire a été sensiblement normal. Le reste de l'examen somatique a été sans particularités. La radiographie thoracique à l'admission est représentée sur la (Figure 1).

### Quel est votre diagnostic?

**Figure 2:**
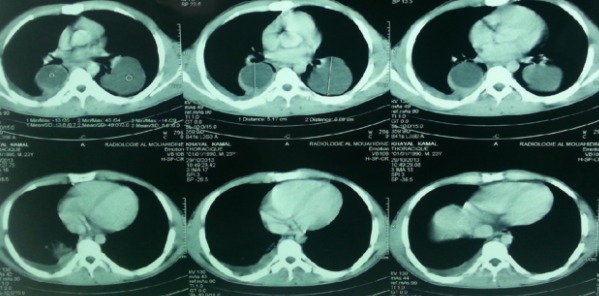
Tomodensitométrie thoracique initiale: aspect en faveur d’un kyste hydatique pulmonaire bilateral

**Figure 3:**
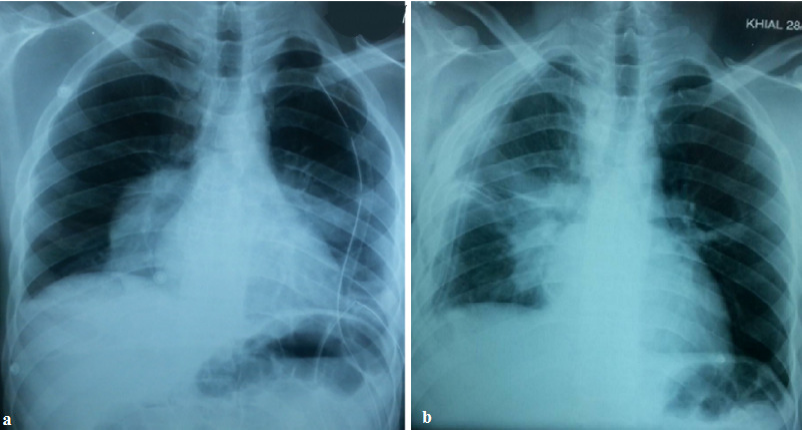
En haut a) radiographie thoracique en postopératoire du kyste hydatique gauche; en bas b) radiographie thoracique en postopératoire du kyste hydatique droit

La radiographie du thorax montre une double opacité à projection hilaire bilatérale grossièrement arrondie à limite nette, une surélévation de la coupole diaphragmatique droite et un émoussement de cul de sac costo-diaphragmatique droit. Devant cet aspect radiologique chez un jeune homme d'un pays méditerranéen, le premier diagnostic à évoquer est celui d'anévrysmes des artères pulmonaires. Cependant, les artères pulmonaires sont bien visibles au sein des opacités et en appliquant le signe de convergence du hile, ces opacités ont peu de chance d'être de nature vasculaire. Dans le même sens, l'interrogatoire et l'examen clinique n'ont pas mis en évidence une aphtose buccale ou génitale ni de cicatrice d'aphtose génitale ou des signes d'uvéite. Une atteinte ganglionnaire médiastinale a été soulevée, d'origine maligne (hémopathie maligne, métastase d'une néoplasie osseuse ou testiculaire), l'examen clinique n'a pas objectivé une atteinte ganglionnaire périphérique ni d'hépato-splénomagalie ni des anomalies en faveur d'une néoplasie notamment testiculaire ou osseuse vu l'âge. Enfin, vu l'origine rurale du patient un kyste hydatique pulmonaire bilatéral était aussi très probable. Le scanner thoracique a montré deux formations kystiques arrondies, homogènes, à paroi fine et nette et sans calcifications avec une pleurésie droite minime sans autres lésions associées (Figure 2). Les coupes passant par le foie ne révélaient pas d'autres lésions kystiques associées qui pouvaient expliquer la surélévation de la coupole diaphragmatique objectivée sur la radiographie thoracique. Cet aspect était en faveur d'un kyste hydatique bilatéral. La sérologie hydatique était positive, la numération formule sanguine a montré une hyperéosinophilie à 990 élément/mm3. L'échographie abdominale n'a pas révélé de kyste hydatique hépatique. La bronchoscopie a montré un état inflammatoire bronchique diffus sans anomalies visibles notamment pas de membranes hydatiques. La recherche dans le liquide d'aspiration bronchique de scolex était négative. Le malade a été opéré d'abord pour le kyste gauche vu sa taille qui était plus grande avec un risque de rupture plus important. L'exploration chirurgicale trouvait une formation kystique au niveau du lobe inférieur gauche contenant du liquide en eau de roche, après son évacuation, un aveuglement de douze fistules bronchiques et un capitonnage de la cavité était réalisé. Trois mois plus tard le patient a été opéré pour le kyste controlatéral avec le même principe (Figure 3).

## Discussion

La maladie hydatique est une infestation parasitaire endémique dans de nombreuses régions d´élevage de moutons et de bovins. Elle est encore un vrai problème de santé publique dans le monde. Le poumon est le deuxième organe le plus touché après le foie, de 10 à 40% selon les études. La fréquence de l'hydatidose pulmonaire bilatérale peut varier de 4 à 26% voire 38% dans certaines régions endémiques [1]. L'imagerie joue un rôle important dans le diagnostic et le bilan d'extension. La radiographie du thorax garde une place importante dans le diagnostic positif, elle permet à elle seule d´affirmer le diagnostic dans près de 90% des cas, dans les formes typiques [2]. La tomodensitométrie permet une étude précise du kyste et du parenchyme périkystique, elle confirme la nature kystique d'une opacité pulmonaire et permet d'éliminer les autres étiologies. Cet examen permet le dénombrement exact des lésions et recherche des anomalies associées telle qu'une dilatation des bronches [3]. Actuellement la stratégie dans la prise en charge chirurgicale de la maladie hydatique pulmonaire est le traitement conservateur. Certains auteurs recommandent d'opérer les polykystoses bilatérales en deux temps avec un intervalle de trois à quatre semaines entre les interventions. Si tous les kystes hydatiques sont intacts, il faut opérer en premier le côté du plus gros kyste, et s'il existe un côté avec un kyste rompu, il faut préférer opérer le côté du kyste intact d'abord car le kyste rompu peut difficilement être aggravé durant le premier temps opératoire. Quand les kystes sont fissurés ou rompus des deux côtés, il faut commencer par opérer d'abord le poumon qui contient le plus de kystes intacts. La chirurgie en un temps peut être faite soit par double thoracotomie, soit par sternotomie médiane afin de diminuer le coût et d'éviter une seconde anesthésie générale. Elle est essentiellement indiquée pour les kystes jeunes, non compliqués et périphériques [1].

## Conclusion

L'hydatidose pulmonaire multiple continue à poser des difficultés diagnostiques étant donné son polymorphisme radio-clinique d'où l'intérêt de réunir un faisceau d'arguments cliniques, sérologiques et radiologiques pour retenir le diagnostic. La chirurgie est le traitement de choix. La diversité du processus pathologique offre différentes tactiques et approches chirurgicales dans le traitement des kystes hydatiques pulmonaires bilatéraux qui doit être adapté individuellement au cas par cas.
